# Comparison of Rapid Antigen Tests for COVID-19

**DOI:** 10.3390/v12121420

**Published:** 2020-12-10

**Authors:** Seiya Yamayoshi, Yuko Sakai-Tagawa, Michiko Koga, Osamu Akasaka, Ichiro Nakachi, Hidefumi Koh, Kenji Maeda, Eisuke Adachi, Makoto Saito, Hiroyuki Nagai, Kazuhiko Ikeuchi, Takayuki Ogura, Rie Baba, Kensuke Fujita, Takahiro Fukui, Fumimaro Ito, Shin-ichiro Hattori, Kei Yamamoto, Takato Nakamoto, Yuri Furusawa, Atsuhiro Yasuhara, Michiko Ujie, Shinya Yamada, Mutsumi Ito, Hiroaki Mitsuya, Norio Omagari, Hiroshi Yotsuyanagi, Kiyoko Iwatsuki-Horimoto, Masaki Imai, Yoshihiro Kawaoka

**Affiliations:** 1 Division of Virology, Department of Microbiology and Immunology, Institute of Medical Science, The University of Tokyo, Tokyo 108-8639, Japan; ytsakai@g.ecc.u-tokyo.ac.jp (Y.S.-T.); kk187321@stu-cbms.k.u-tokyo.ac.jp (Y.F.); yasuhara@ims.u-tokyo.ac.jp (A.Y.); m-ujie@ims.u-tokyo.ac.jp (M.U.); syamada@g.ecc.u-tokyo.ac.jp (S.Y.); ito-mu@ims.u-tokyo.ac.jp (M.I.); kenken@ims.u-tokyo.ac.jp (K.I.-H.); mimai@ims.u-tokyo.ac.jp (M.I.); 2 Division of Infectious Diseases, Advanced Clinical Research Center, Institute of Medical Science, The University of Tokyo, Tokyo 108-8639, Japan; michiko@ims.u-tokyo.ac.jp (M.K.); saito-id@ims.u-tokyo.ac.jp (M.S.); kikeuchi004@gmail.com (K.I.); yotsudid@ims.u-tokyo.ac.jp (H.Y.); 3 Department of Infectious Diseases and Applied Immunology, IMSUT Hospital of Institute of Medical Science, The University of Tokyo, Tokyo 108-8639, Japan; e-adachi@ims.u-tokyo.ac.jp (E.A.); nagai.hir@gmail.com (H.N.); 4 Emergency Medical Center, Fujisawa City Hospital, Kanagawa 251-8550, Japan; samukun036@gmail.com; 5 Pulmonary Division, Department of Internal Medicine, Saiseikai Utsunomiya Hospital, Tochigi 321-0974, Japan; nichiro4747@gmail.com (I.N.); rie.amechan@gmail.com (R.B.); 6 Division of Pulmonary Medicine, Department of Internal Medicine, Tachikawa Hospital, Tokyo 190-8531, Japan; h.koh@tachikawa-hosp.gr.jp (H.K.); ftakahiro0529@yahoo.co.jp (T.F.); fito102351@gmail.com (F.I.); 7 Department of Refractory Viral Infections, National Center for Global Health and Medicine Research Institute, Tokyo 162-8655, Japan; kmaeda@ri.ncgm.go.jp (K.M.); shattori@ri.ncgm.go.jp (S.-i.H.); hmitsuya@hosp.ncgm.go.jp (H.M.); 8 Department of Emergency Medicine and Critical Care Medicine, Saiseikai Utsunomiya Hospital, Tochigi 321-0974, Japan; alongthelongestway2003@yahoo.co.jp (T.O.); fujitak.727@gmail.com (K.F.); 9 Disease Control and Prevention Center, National Center for Global Health and Medicine Hospital, Tokyo 162-8655, Japan; kyamamoto@hosp.ncgm.go.jp (K.Y.); tnakamoto@hosp.ncgm.go.jp (T.N.); nohmagari@hosp.ncgm.go.jp (N.O.); 10 Department of Pathobiological Sciences, School of Veterinary Medicine, University of Wisconsin-Madison, Madison, WI 53706, USA; 11 International Research Center for Infectious Diseases, Department of Special Pathogens, Institute of Medical Science, The University of Tokyo, Tokyo 108-8639, Japan

**Keywords:** rapid antigen test, diagnosis, COVID-19, SARS-CoV-2

## Abstract

Reverse transcription-quantitative PCR (RT-qPCR)-based tests are widely used to diagnose coronavirus disease 2019 (COVID-19). As a result that these tests cannot be done in local clinics where RT-qPCR testing capability is lacking, rapid antigen tests (RATs) for COVID-19 based on lateral flow immunoassays are used for rapid diagnosis. However, their sensitivity compared with each other and with RT-qPCR and infectious virus isolation has not been examined. Here, we compared the sensitivity among four RATs by using severe acute respiratory syndrome coronavirus 2 (SARS-CoV-2) isolates and several types of COVID-19 patient specimens and compared their sensitivity with that of RT-qPCR and infectious virus isolation. Although the RATs read the samples containing large amounts of virus as positive, even the most sensitive RAT read the samples containing small amounts of virus as negative. Moreover, all RATs tested failed to detect viral antigens in several specimens from which the virus was isolated. The current RATs will likely miss some COVID-19 patients who are shedding infectious SARS-CoV-2.

## 1. Introduction

Severe acute respiratory syndrome coronavirus 2 (SARS-CoV-2), which emerged as a novel human pathogen in China at the end of 2019 [1], is responsible for coronavirus disease 2019 (COVID-19), which causes symptoms such as cough and fever, severe pneumonia, and death. The WHO reported that more than 29 million cases of COVID-19, including approximately 900,000 deaths, have occurred as of 16 September 2020 (https://covid19.who.int/). To control the spread of SARS-CoV-2 infections, rapid identification and isolation of patients are required.

The gold standard for COVID-19 diagnosis is reverse transcription-quantitative PCR (RT-qPCR) using nasopharyngeal (N) swabs, throat (T) swabs, or saliva [2]. RT-qPCR kits that do not require viral RNA extraction and high-throughput RT-qPCR systems have been developed. Although such tests are widely utilized in public health laboratories and large well-equipped hospitals, they are unavailable in local clinics where patients who suspect they have COVID-19 often go first. Therefore, specimens need to be transported to and examined at sites that have RT-qPCR capability, which delays the test result and increases the anxiety of the suspected COVID-19 patients. To improve this situation, rapid antigen tests (RATs) for COVID-19, which does not require specific and expensive machinery, have been approved for clinical use in Japan and other countries, and the sensitivity of these tests has been compared with that of several kinds of RT-qPCR [3,4,5,6,7,8,9]. Although these RATs might be useful for the identification of COVID-19 patients in local clinics, their sensitivity is important in determining usage strategies. Here, we examined the sensitivity of four RATs available in Japan in August 2020 for the detection of isolated viruses. We also evaluated their effectiveness with several kinds of clinical specimens collected from COVID-19 patients and compared it with that of RT-qPCR and virus isolation.

## 2. Materials and Methods

### 2.1. Ethics and Biosafety Statements

Human samples were collected by following protocols approved by the Research Ethics Review Committee of the Institute of Medical Science, the University of Tokyo (approval number 2019-71-0201; 1 February 2020). Signed informed consent was obtained from all participants.

All experiments with SARS-CoV-2 were performed prior to 8 September 2020 in biosafety level 3 (BSL3) laboratories at the University of Tokyo, which were approved for such use by the Ministry of Agriculture, Forestry, and Fisheries, Japan.

### 2.2. Cells

Vero cells expressing human serine protease TMPRSS2 (Vero-TMPRSS2) [10] were maintained in DMEM containing 10% fetal calf serum (FCS), 1 mg/mL G418, 100 units/mL penicillin, 100 µg/mL streptomycin, and 5 μg/mL plasmocin prophylactic (InvivoGen, San Diego, CA, USA) and incubated at 37 °C under 5% CO_2_. We used these cells after clearance of mycoplasma.

### 2.3. Viruses

SARS-CoV-2 (UT-NCGM02/Human/2020/Tokyo (NC02) and UT-HP072/Human/2020/Tokyo (HP72)] were isolated from clinical samples collected in February and August, respectively, and titrated in Vero-TMPRSS2 cells by performing plaque assays [11].

### 2.4. Clinical Samples

Gargle lavage (*n* = 7), saliva (*n* = 27), throat (T) swab (*n* = 2), nasal vestibule swab (*n* = 1), nasopharyngeal (*N*) swab (*n* = 18), sputum (*n* = 4), and tracheal aspirate (*n* = 17) samples (Supplementary Materials Table S1) were collected from COVID-19 patients at several timepoints after onset. Swabs were soaked in BD universal viral transport medium; saliva, sputum, and tracheal aspirate samples were diluted in BD universal viral transport medium if needed. These media were used as test specimens. Gargle lavages were examined directly.

### 2.5. RT-qPCR

Viral RNA was isolated from the specimens by using the QIAamp Viral RNA Mini Kit (QIAGEN, Tokyo, Japan). One step RT-qPCR was performed using the LightCycler 96 System (Roche Diagnostics, Tokyo, Japan) according to the protocol of the National Institute of Infectious Disease, Japan [12]. A Cq value of >40 was considered a negative result.

### 2.6. Rapid Antigen Test (RAT)

The RATs listed in Table 1 were evaluated according to the procedures described in the manufacturers’ instructions, using 75–7500 plaque formation unit (PFU) of stock virus in 50 μL of culture supernatant or 50 μL of specimen. Two independent experiments were performed with each sample.

### 2.7. Virus Isolation

The specimens were inoculated into Vero-TMPRSS2 cells in 24 well-plates and the cells were incubated at 37 °C for 1 h. After removal of the inoculum, the cells were incubated in DMEM containing 5% FCS, 10 mM HEPES, 100 μg/mL gentamicin sulfate, and 2.5 μg/mL amphotericin B for 6 days at 37 °C. The appearance of cytopathic effects was checked for 6 days at least once a day.

## 3. Results

### 3.1. Comparison of 4 Rapid Antigen Tests (RATs)

We evaluated four RATs that were available in Japan in August 2020 (Table 1). These RATs are immunochromatographic tests; therefore, their sensitivity is dependent on the binding kinetics of the monoclonal antibodies used in each RAT. The composition of the lysis buffer, the proportion of specimen in the analyte, and the method used to visualize the result also affect the sensitivity. As a result that the manufacturers do not disclose the composition of the lysis buffer, we could not compare this parameter. The amount of lysis buffer and specimen/lysis buffer mixture used for testing differed among the RATs tested. Accordingly, the percentages of the specimens used also differed: for Standard Q COVID-19 Ag, Espline SARS-CoV-2, QuickNavi -COVID19 Ag, and ImmunoAce SARS-CoV-2, the percentages of specimens used were 14.3%, 10.0%, 12.5%, and 28.6%, respectively (Table 1). The method for visualization of the results also differed among these tests in that Standard Q COVID-19 Ag, Espline SARS-CoV-2, QuickNavi -COVID19 Ag, and ImmunoAce SARS-CoV-2 use color particles, alkaline phosphatase and its substrate, color latex, and platinum-gold colloids, respectively, to visualize the antigen-antibody immune-complexes. The visualized results are assessed by the human eye at 15–30, 30, 15, and 15 min, respectively, after adding the analyte. Although the types of specimens recommended for all four RATs are N swabs, we tested other types of specimens (see the footnote to Table 2 and Table 3).

### 3.2. Sensitivity of RATs for Two Isolated SARS-CoV-2 Strains

To compare the sensitivity of these four RATs, two SARS-CoV-2 isolate stocks (NC02 and HP72) were diluted to the indicated PFU and then examined by RT-qPCR to determine the Cq value of each sample. The Cq values were 18.0, 21.7, 22.2, 23.1, and 25.4 at 7500, 750, 500, 250, and 75 PFU of NC02 and 16.9, 17.8, 18.8, 20.4, 21.1, 22.7, and 24.0 at 7500, 5000, 2500, 750, 500, 250, and 75 PFU of HP72 (Table S2). Standard Q COVID-19 Ag and ImmunoAce SARS-CoV-2 detected as little as 250 PFU of NC02, whereas Espline SARS-CoV-2 and QuickNavi -COVID19 Ag detected 500 PFU and 750 PFU of NC02, respectively (Table S2). Standard Q COVID-19 Ag and Espline SARS-CoV-2 detected as little as 250 PFU of HP72, whereas QuickNavi COVID19 Ag and ImmunoAce SARS-CoV-2 detected 5000 PFU of HP72. These results show that the sensitivity for virus detection of Standard Q COVID-19 Ag and Espline SARS-CoV-2 was better than that of QuickNavi COVID19 Ag, and that virus detection by ImmunoAce SARS-CoV-2 differed depending on the isolate.

### 3.3. Sensitivity of RATs for Clinical Specimens

To evaluate the sensitivity of the four RATs for clinical specimens, 7 gargle lavages, 27 saliva, 2 throat (T) swabs, 1 nasal vestibule swabs, 18 nasopharyngeal (N) swabs, 4 sputum, and 17 tracheal aspirates were collected and their viral genomic RNA levels were examined by RT-qPCR. These 76 clinical specimens, which were divided into 6 groups based on their Cq value, were examined in the four RATs and subjected to virus isolation using Vero-TMPRSS2 cells (Table 2 and Table S1). Standard Q COVID-19 Ag and Espline SARS-CoV-2 detected viral antigens in all specimens with Cq values lower than 22.5, whereas QuickNavi COVID19 Ag and ImmunoAce SARS-CoV-2 failed to detect viral antigens in several of these specimens. For specimens with a Cq value between 22.5 and 25.0, the sensitivity was on the order of Espline SARS-CoV-2, Standard Q COVID-19 Ag, ImmunoAce SARS-CoV-2, and QuickNavi COVID19 Ag (Table 2 and Table S1). For the specimens with a Cq value greater than 25.0, the sensitivity of these four RATs was similar. These results indicate that the sensitivity of the four RATs is lower than that of RT-qPCR but similar to that of virus isolation. Overall, the sensitivity of Espline SARS-CoV-2 and Standard Q COVID-19 Ag was slightly better than that of ImmunoAce SARS-CoV-2; QuickNavi -COVID19 Ag had the lowest sensitivity.

**Figure 1:**
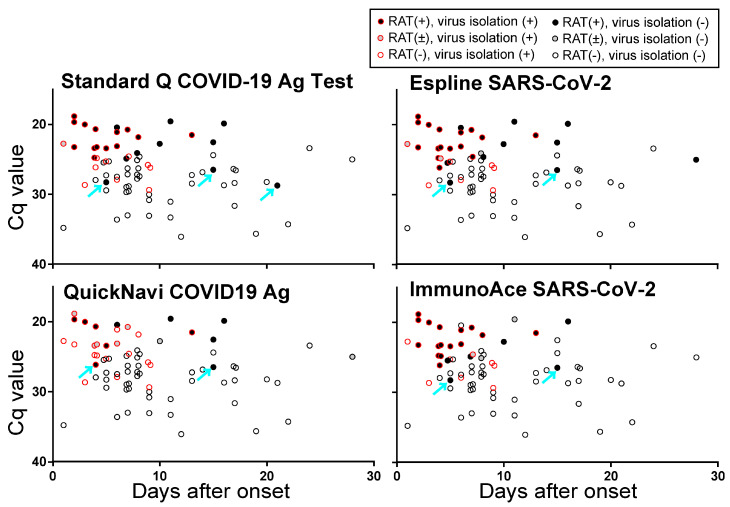
Results of RATs plotted based on the Cq value of RT-qPCR and days after onset. Viral antigens in 76 clinical specimens (7 gargle lavages, 27 saliva, 2 T swabs, 1 nasal vestibule swabs, 18 N swabs, 4 sputum, and 17 tracheal aspirates) were examined by Standard Q COVID-19 Ag, Espline SARS-CoV-2, QuickNavi COVID19 Ag, and ImmunoAce SARS-CoV-2. Two independent experiments were performed. All specimens were also tested by RT-qPCR and subjected to virus isolation. Open red circles indicate specimens from which virus was isolated; open black circles indicate specimens from which virus was not isolated. Circles filled in black indicate both results were positive, those filled in gray indicate that one was positive, no color indicates both were negative.

We next plotted the results from the RATs and virus isolation, in accordance with the number of days after onset that the specimens were obtained, and the Cq values of the RT-qPCR (Figure 1). Although all of the RATs efficiently detected viral antigens in the specimens with low Cq values, the results from the RATs could be different for two specimens with similar Cq values (i.e., some were RAT-positive, whereas others were RAT-negative). Several specimens with Cq values higher than those of specimens that were RAT-negative, but with the above-described low Cq values (as indicated by cyan arrows in Figure 1) were found to be RAT-positive. These results suggest that the viral RNA and protein content may have differed between the specimens or that some samples may have contained inhibitors or enhancers for the antigen-antibody reaction of the RATs. Importantly, several specimens from which the virus was isolated were negative with all RATs tested, indicating that these RATs could potentially fail to diagnose patients shedding infectious SARS-CoV-2.

**Figure 2:**
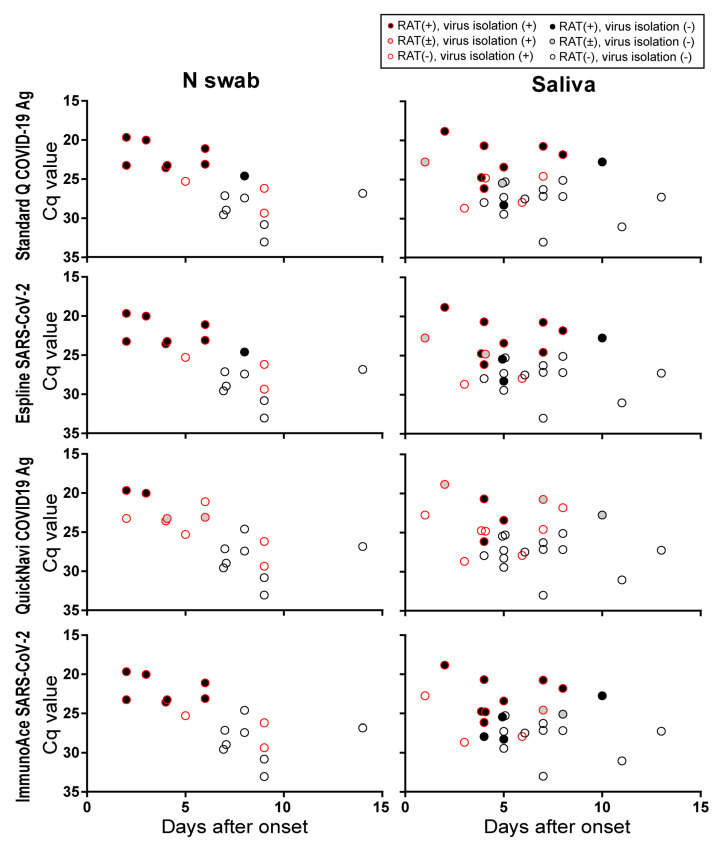
Results of RATs using N swabs and saliva specimens plotted based on the Cq value of RT-qPCR and days after onset. The results from the N swabs and saliva samples were extracted from Figure 1.

Since the type of specimen likely affects the sensitivity of RATs, we assessed our results according to specimen type (gargle lavage, saliva, T swab, nasal vestibule swab, N swab, sputum, and tracheal aspirate). Although the Cq value varied between specimens from 18.8 to 36.0, gargle lavage, T swabs, and nasal vestibule swabs showed higher Cq values than the other types of specimens (Table 3). All four RATs detected virus antigens in one sputum sample, although no virus was isolated from 4 sputum samples tested. All four RATs failed to detect virus antigens in all 7 gargle lavages, 2 T swabs, and 1 nasal vestibule swabs, although the virus was isolated from one T swab sample. Since N swabs and saliva are suitable specimens for diagnosis in clinics, we plotted the results from the RATs and virus isolation based on the number of days after onset that the specimens were collected and the Cq values of the RT-qPCR (Figure 2). Standard Q COVID-19 Ag, Espline SARS-CoV-2, and ImmunoAce SARS-CoV-2 efficiently detected virus antigens in N swabs and saliva with low Cq values (Cq < 25) that were collected at early timepoints after onset.

## 4. Discussion

Here we evaluated the sensitivity of four RATs available in Japan in August 2020. The overall sensitivity of Standard Q COVID-19 Ag and Espline SARS-CoV-2 was better than that of ImmunoAce SARS-CoV-2 and QuickNavi COVID19 Ag. For specimens such as saliva and swabs, Standard Q COVID-19 Ag, Espline SARS-CoV-2, and ImmunoAce SARS-CoV-2 had similar detection sensitivities. These three kits detected viral antigens in more than half of the specimens whose Cq values of RT-qPCR were less than approximately 25. Therefore, these RATs may be suitable for the detection of COVID-19 in individuals who are shedding a large amount of SARS-CoV-2; that is, they may be useful to identify patients with a high likelihood of transmitting the virus to others.

All four RATs failed to detect viral antigens in several specimens from which virus was isolated. This finding indicates that the current RATs are likely to miss some COVID-19 patients who are shedding infectious SARS-CoV-2. However, it is unclear to what extent such patients would transmit virus to others. Further studies are required to address this point. In addition, the present study used swab specimens that were soaked in transport medium; if the specimens were directly soaked in lysis buffer, the sensitivity of the RATs might be improved. The recommended samples for these RATS are nasopharyngeal swabs not saliva, and some of the saliva samples from which SARS-CoV-2 was isolated gave negative results with all of the RATs tested here. Nonetheless, these RATs need to be improved with respect to their sensitivity.

In this study, we focused on the sensitivity of four RATs; we did not evaluate the false-positive rates of these kits. Therefore, studies on false-positive rates should be performed in the future to determine the specificity of these and other RATs.

## Figures and Tables

**Table 1:** Rapid antigen tests examined in this study.

Name	Manufacturer	Method for Visualization ^a^	Input Ratio ^b^ (%)	Minutes to Assess ^c^	Country of Manufacture
Standard Q COVID-19 Ag	SD Biosensor	Color particle	14.3	15–30	Korea
Espline SARS-CoV-2	Fujirebio	Alkaline phosphatase	10.0	30	Japan
QuickNavi -COVID19 Ag	Denka Seiken	Color latex	12.5	15	Japan
ImmunoAce SARS-CoV-2	Tauns Laboratories	Platinum-gold colloid	28.6	15	Japan

^a^ The lysed sample is dropped into the well and the reaction occurs inside a covered plastic body. ^b^ For all rapid antigen tests (RATs) tested, the test samples (50 μL) were mixed with lysis buffer (A). A part of the lysed sample (B) was then assayed. Input ratios were calculated using the following formula: volume B/(50 μL + volume A) × 100. ^c^ The time required to obtain the results is based on the manufacturer’s instructions provided with the kit.

**Table 2:** Sensitivity of rapid antigen tests for clinical specimens.

RT-qPCR	Number of Samples ^a^	Standard Q COVID-19 Ag	Espline SARS-CoV-2	QuickNavi COVID19 Ag	ImmunoAce SARS-CoV-2	Virus Isolation
(Cq Value)						
–20.0	4	4 ^b^	4	3.5	3.5	2 ^c^
20.0–22.5	7	7	7	4.5	6	6
22.5–25.0	17	10.5	12.5	5	9.5	9
25.0–27.5	20	2.5	3	2	3.5	4
27.5–30.0	17	2	1	0	2	3
30.0–	11	0	0	0	0	0

^a^ A total of 76 clinical specimens including 7 gargle lavages, 27 saliva, 2 T swabs, 1 nasal vestibule swabs, 18 N swabs, 4 sputum, and 17 tracheal aspirates were examined. ^b^ Each sample was tested in two independent experiments; when a kit identified a sample as positive in both tests, a value of 1 was assigned. If only one test was positive, a value of 0.5 was assigned. If both tests were negative, a value of 0 was assigned. ^c^ Number of samples from which the virus was isolated.

**Table 3:** Sensitivity of rapid antigen tests for different types of clinical specimen.

Clinical Specimen	Number of Samples	RT-qPCR	Standard Q COVID-19 Ag	Espline SARS-CoV-2	QuickNavi COVID19 Ag	ImmunoAce SARS-CoV-2	Virus Isolation
		(Cq Value)					
Gargle lavage	7	26.3–36.0	0 ^a^	0	0	0	0 ^b^
Saliva	27	18.8–33.0	10	12	5	13	12
T Swab	2	**25.8**^c^, 33.6	0	0	0	0	1
Nasal vestibule swab	1	34.8	0	0	0	0	0
N swab	18	19.7-33.0	8	8	3.5	7	10
Sputum	4	19.9-34.3	1	1	1	1	0
Tracheal aspirate	17	19.6-35.6	7	6.5	5.5	3.5	1

^a^ Each specimen was tested in two independent experiments; when a kit identified a sample as positive in both tests, a value of 1 was assigned. If only one test was positive, a value of 0.5 was assigned. If both tests were negative, a value of 0 was assigned. ^b^ Number of samples from which virus was isolated. ^c^ Virus was isolated from this specimen.

## Supplementary Materials

The following are available online at https://www.mdpi.com/1999-4915/12/12/1420/s1, Table S1: List of specimens and results, Table S2: Sensitivity of rapid antigen tests for stock viruses.
