# Supplementary material for: Comparison of Rapid Antigen Tests for COVID-19
Source: Viruses. 2020 Dec 10;12(12):1420. doi: 10.3390/v12121420 (PMC7764512; doi:10.3390/v12121420)
Supplement: Supplementary file 1 [file viruses-12-01420-s001.pdf]

Table S1. List of specimens and results

ID	Specimen	Days post-onset sample obtained	Cq value of RT-qPCR	Standard Q COVID-19 Ag	Espline SARS-CoV-2	QuickNavi -COVID19 Ag	ImmunoAce SARS-CoV-2	Infectious virus isolation
SUCo-015	Nasal vestibule swab	1	34.8	-	-	-	-	-
HPCo-049	Saliva	1	22.7	±	±	-	-	+
HPCo-040	N swab	2	23.2	+	+	-	+	+
SUCo-014	N swab	2	19.7	+	+	+	+	+
HPCo-070	Saliva	2	18.8	+	+	±	+	+
HPCo-038	Saliva	3	28.7	-	-	-	-	+
HPCo-038	N swab	3	20.0	+	+	+	+	+
HPCo-071	Saliva	4	27.9	-	-	-	+	-
HPCo-070	Saliva	4	26.1	+	+	+	+	+
HPCo-050	Saliva	4	24.8	-	±	-	+	+
HPCo-038	Saliva	4	24.7	+	+	-	+	+
HPCo-049	N swab	4	23.4	+	+	±	+	+
HPCo-053	N swab	4	23.2	+	+	±	+	+
TKCo-007	Saliva	4	20.7	+	+	+	+	+
HPCo-038	Saliva	5	29.4	-	-	-	-	-
HPCo-070	Saliva	5	28.3	+	+	-	+	-
HPCo-057	Saliva	5	27.3	-	-	-	-	-
HPCo-073	Saliva	5	25.4	±	+	-	+	-
HPCo-054	Saliva	5	25.3	-	-	-	-	-
HPCo-054	N swab	5	25.3	-	-	-	-	+

TKCo-007	Saliva	5	23.4	+	+	+	+	+
NCCo-001	T Swab	6	33.6	-	-	-	-	-
HPCo-056	Saliva	6	27.9	-	-	-	-	+
TKCo-007	Saliva	6	27.5	-	-	-	-	-
SUCo-007	N swab	6	23.1	+	+	±	+	+
HPCo-040	N swab	6	21.1	+	+	-	+	+
FSCo-001	Tracheal aspirate	6	20.4	+	+	+	-	-
TKCo-007	Saliva	7	33.0	-	-	-	-	-
HPCo-016	Gargle lavage	7	29.7	-	-	-	-	-
SUCo-006	N swab	7	29.6	-	-	-	-	-
HPCo-049	N swab	7	29.0	-	-	-	-	-
TKCo-001	Gargle lavage	7	28.8	-	-	-	-	-
HPCo-056	Saliva	7	27.1	-	-	-	-	-
HPCo-053	N swab	7	27.1	-	-	-	-	-
HPCo-073	Saliva	7	26.3	-	-	-	-	-
FSCo-007	Tracheal aspirate	7	24.9	+	-	-	+	-
HPCo-070	Saliva	7	24.6	-	+	-	±	+
TKCo-010	Saliva	7	20.7	+	+	±	+	+
FSCo-005	Tracheal aspirate	8	27.8	-	-	-	-	-
HPCo-056	N swab	8	27.4	-	-	-	-	-
HPCo-058	Saliva	8	27.2	-	-	-	-	-
TKCo-001	Gargle lavage	8	26.3	-	-	-	-	-
HPCo-050	Saliva	8	25.1	-	-	-	±	-
HPCo-042	N swab	8	24.6	+	+	-	-	-
FSCo-001	Tracheal aspirate	8	24.1	-	±	-	-	-

HPCo-070	Saliva	8	21.8	+	+	-	+	+
SUCo-017	N swab	9	33.0	-	-	-	-	-
HPCo-042	N swab	9	30.8	-	-	-	-	-
HPCo-015	Gargle lavage	9	30.0	-	-	-	-	-
HPCo-054	N swab	9	29.4	-	-	-	-	+
HPCo-038	N swab	9	26.2	-	-	-	-	+
NCCo-002	T Swab	9	25.8	-	-	-	-	+
HPCo-070	Saliva	10	22.8	+	+	±	+	-
HPCo-015	Gargle lavage	11	33.3	-	-	-	-	-
HPCo-058	Saliva	11	31.0	-	-	-	-	-
FSCo-004	Tracheal aspirate	11	19.6	+	+	+	±	-
HPCo-015	Gargle lavage	12	36.0	-	-	-	-	-
FSCo-004	Tracheal aspirate	13	28.4	-	-	-	-	-
HPCo-074	Saliva	13	27.2	-	-	-	-	-
FSCo-003	Tracheal aspirate	13	21.5	+	+	+	+	+
HPCo-051	N swab	14	26.8	-	-	-	-	-
FSCo-006	Tracheal aspirate	15	26.5	+	+	+	+	-
FSCo-004	Tracheal aspirate	15	24.4	-	-	-	-	-
FSCo-003	Tracheal aspirate	15	22.5	+	+	+	-	-
HPCo-015	Gargle lavage	16	28.7	-	-	-	-	-
SUCo-002	Sputum	16	19.9	+	+	+	+	-
FSCo-003	Tracheal aspirate	17	31.6	-	-	-	-	-
FSCo-006	Tracheal aspirate	17	28.4	-	-	-	-	-
FSCo-004	Tracheal aspirate	17	26.6	-	-	-	-	-
FSCo-002	Tracheal aspirate	17	26.3	-	-	-	-	-

FSCo-004	Tracheal aspirate	19	35.6	-	-	-	-	-
SUCo-002	Sputum	20	28.2	-	-	-	-	-
FSCo-004	Tracheal aspirate	21	28.7	+	-	-	-	-
SUCo-004	Sputum	22	34.3	-	-	-	-	-
SUCo-002	Sputum	24	23.4	-	-	-	-	-
FSCo-001	Tracheal aspirate	28	25.0	-	+	±	-	-

Table S2. Sensitivity of rapid antigen tests for stock viruses

Test	Virus titer of NC02[a] tested (PFU)					Virus titer of HP72[b] tested (PFU)						
	7500	750	500	250	75	7500	5000	2500	750	500	250	75
RT-qPCR	18.0[c]	21.7	22.2	23.1	25.4	16.9	17.8	18.8	20.4	21.1	22.7	24.0
Standard Q COVID-19 Ag	+[d]	+	+	+	-	+	n.d.[e]	n.d.	+	+	+	-
Espline SARS-CoV-2	+	+	+	-	-	+	n.d.	n.d.	+	+	+	-
QuickNavi -COVID19 Ag	+	+	-	-	-	+	+	-	-	n.d.	n.d.	-
ImmunoAce SARS-CoV-2	+	+	+	+	-	+	+	-	-	n.d.	n.d.	-

UT-NCGM02/Human/2020/Tokyo[a] and UT-HP072/Human/2020/Tokyo[b] were examined with each RAT according to the manufacturers' instructions.

[c]Average Cq value of RT-qPCR (n=2).

[d]Two independent experiments were performed: '+' indicates both were positive, '-' indicates both were negative.

[e]Not done.
